# Attributes Desired in a General Practitioner by Adults: Implications for Humanization in Medicine and Enhancement of the Patient Experience

**DOI:** 10.3390/healthcare12242589

**Published:** 2024-12-23

**Authors:** Agnieszka J. Szczepek, Malgorzata Wrzosek, Marta Makowska

**Affiliations:** 1 Department of Otorhinolaryngology, Head and Neck Surgery, Charité-Universitätsmedizin Berlin, Corporate Member of Freie Universität Berlin and Humboldt-Universität zu Berlin, 10117 Berlin, Germany; 2 Faculty of Medicine and Health Sciences, University of Zielona Góra, 65-046 Zielona Góra, Poland; 3 Faculty of Psychology and Cognitive Science, Adam Mickiewicz University, 60-568 Poznań, Poland; mwrzosek@amu.edu.pl; 4 Department of Economic Psychology, Kozminski University, 03-301 Warszawa, Poland; mmakowska@kozminski.edu.pl

**Keywords:** medical humanities, humanization of medicine, patient–physician relationship, patient–physician communication, general practitioner, patients’ expectations, enhancement in patient experience

## Abstract

Background: Progress in medical education is reflected in healthcare quality and patient satisfaction. However, there are still gaps in knowledge regarding the patients’ expectations of general practitioners (GPs), even though patients’ interactions with GPs are among the most frequent in healthcare. Accordingly, this study investigated the qualities desired by adults in GPs, intending to include them in future medical humanization education to enhance the patients’ experience. Methods: Using an online survey, 1057 respondents (52.8% female; mean age, 46.55 years old, SD 16.03) were asked to rate 15 statements on a scale of 1 to 10 regarding desirable characteristics of general practitioners. In addition to the respondents’ high regard for their GP’s ongoing education and sincerity in conveying both positive and negative information, the findings suggest that the socio-demographic attributes of the respondents influence their expectations of GPs. Results: Women had higher overall expectations than men. In addition, those who reported higher satisfaction with their lives and recent healthcare users had higher expectations of GPs than those who were dissatisfied. The youngest group of respondents exhibited the lowest expectations of the GPs’ attributes, except for their greater willingness to engage in discussions with GPs regarding personal or professional issues, compared with the older groups. Conclusions: The information presented in this work can be utilized during medical students’ humanization training and physicians’ continuing education. The knowledge gained should enhance GPs’ awareness of the distinctions in patients’ expectations, enabling them to adapt their approach and services to align with their patients’ unique needs, expectations, and experiences.

## 1. Introduction

In recent decades, medical education in many countries has incorporated courses intending to humanize healthcare [1,2]. These efforts are not limited to the education of physicians but also extend to that of nurses [3,4,5] or paramedics [6,7]. These subjects, commonly referred to as medical humanities, encompass a range of disciplines, including medical sociology, psychology, the history of medicine, and patients’ rights, among others [5]. The primary objective of incorporating these courses into the curriculum is to shape future healthcare professionals who are knowledgeable in their field and attuned to the nuances of human suffering and the needs of the sick. It also aims to acquire essential communication skills when interacting with patients and their families. The standards for teaching medical humanities are informed by scientific research and the professional experience of healthcare professionals, highlighting the crucial role of this subject in shaping the future of patient care.

The Polish healthcare system is based on social health insurance (Narodowy Fundusz Zdrowia, NFZ), providing primary care services free of charge, including to individuals lacking NFZ coverage. In addition to the NFZ, supplementary health insurance covers several types of private healthcare. GPs serve as the initial point of contact for patients, assuming a pivotal role in the management of both acute and chronic conditions. While access to primary care is generally adequate, patients residing in rural areas encounter more significant obstacles to care than their urban counterparts. Generally, general practitioners (GPs) act as gatekeepers, referring patients to specialized care when appropriate. However, the efficacy of primary care services in Poland is significantly compromised by many systemic challenges, including workforce shortages and suboptimal organizational solutions [8].

While the curriculum of healthcare professionals emphasizes the humanizing aspects of medical care, there is a significant gap in evidence indicating their awareness of their patients’ needs in this regard. Most of the data on this topic originate from patients undergoing treatment for terminal illnesses [4,5] or seeking medical assistance within hospitals or outpatient facilities [7]. In contrast, studies addressing patients’ expectations of general practitioners (GPs) or their relationships are scarce. One such study, by Lee et al., sought to ascertain the relevance of the Miller–Coulson Academy of Clinical Excellence (MCACE) application in evaluating the quality of work performed by GPs. The researchers reviewed over two thousand case reports, selected eight, and concluded that MCACE can be used to score clinical excellence, including professionalism, communication, and humanities in primary care [9]. A recent study by Wang et al. employed a qualitative approach to ascertain a definition of a “good doctor”. The study concluded that patients emphasize the following aspects of their healthcare providers: clinical competency, professionalism and humanism, service provision, communication of information, and personal characteristics [10]. Nevertheless, although contact with a general practitioner represents the most frequent point of contact in the healthcare system, there is a lack of information regarding patients’ specific expectations of their doctors regarding humanization. This may result in discrepancies between the content of the humanizing themes learned by all students and actual clinical scenarios. It seems reasonable to postulate that patients with severe or terminal illnesses have different needs and expectations from those with less severe conditions, such as a common cold, when seeking care from a primary care provider.

This raises novel and crucial questions. What expectations do the latter type of patient have regarding the general practitioner’s behavior toward them? Do patients hold such expectations at all? Are there any differences based on age, gender, or state of health? In light of these questions, we undertook a study to investigate this issue among adults of both sexes, representing various age groups, professional categories, educational backgrounds, marital statuses, frequencies of healthcare utilization, and other demographic characteristics. In their daily practice, GPs must interact with individuals from diverse socio-demographic backgrounds, including differences in education, financial status, and generational differences. These factors may influence how they communicate with each person, including their language and gestures. Therefore, studying people with diverse characteristics similar to those that physicians encounter is essential.

The principal objective of this study was to ascertain whether the general population has well-defined expectations of general practitioners concerning the skills associated with humanization acquired during medical training (e.g., communication skills, medical sociology, psychology [11]). In this way, the study makes a significant contribution to the fields of medicine and health sciences, as well as social communication and media studies. It addresses critical components of interpersonal communication within healthcare, particularly in GP–patient interactions. The present paper investigates the specific qualities Polish patients desire in their GPs, focusing on the humanistic aspects of care, such as empathy, sincerity, and effective communication, which are integral to establishing trust and effective patient–physician relationships.

## 2. Materials and Methods

### 2.1. Study Sample

The study sample was selected based on inclusion criteria (age over 18 and internet literacy) and exclusion criteria (age under 18, unwillingness to provide the requested socio-demographic data, completion of the survey in an inordinate amount of time indicating a lack of attention to the questions, and failure to complete the survey). The final sample represented quotas reflecting the Polish population’s age, gender, education (higher vs. other), province, and residence size. Table 1 shows detailed characteristics of the sample.

### 2.2. Survey Administration

The survey was conducted by Ariadna, one of Poland’s most extensive internet panels (https://panelariadna.com/ accessed on 19 December 2024). Ariadna employs a points-based rewards system whereby respondents can earn points by completing surveys. The respondents were at liberty to choose whether or not to participate in the study after receiving an invitation link from the panel. Initially, the respondents were asked to qualify for the survey by indicating their gender and age. Individuals who did not meet the specified criteria were awarded one reward point for participating in the survey. In contrast, respondents who met the criteria were granted 15 reward points for completing the survey. Subsequently, the accumulated points could be redeemed for rewards selected from the reward pool, following the specifications outlined. The survey was open for five days between the 16th and 19th of May 2023.

The mean time required for respondents to complete the survey was approximately 12 min.

### 2.3. Survey Design

The survey questionnaire concentrated on three principal areas: expectations of GPs, issues about trust and collaboration between pharmaceutical companies and GPs, and issues related to collaboration and trust between pharmaceutical companies and patient organizations. This study was part of a larger research project investigating patients’ expectations regarding the ethical conduct, professional behavior, and transparency of general practitioners’ business practices. Nevertheless, the subsequent analysis and reflection led us to concentrate on the specific theme presented in this article, which is connected to the humanization of medicine rather than the pharmaceutical industry.

A preliminary survey was conducted with 12 individuals whose feedback was duly considered. This pilot study allowed us to identify potential issues related to the questions’ clarity, response patterns, and overall structure. Feedback from participants was incorporated into subsequent revisions to improve the comprehensibility and relevance of the questions.

Expert consultation was also required to ensure the questionnaire’s validity. We consulted professionals with extensive experience in survey design and implementation, including Public Opinion Research (CBOS) and National Research Panel Ariadna researchers. Their input was critical in evaluating the questionnaire’s structure, wording, and suitability for the target population. The involvement of these experts ensured that the questionnaire met high methodological standards, including clarity, relevance, and alignment with best practices in survey design.

The final version of the questionnaire was designed following a thorough review of the pilot study’s results. The questions were refined to align with this study’s objectives, ensuring they were concise and focused on capturing the intended constructs.

Ultimately, the questionnaire comprised 6 primary and 16 secondary questions. The following article presents an analysis of the results of one of the main areas (expectations of GPs) concerning various socio-demographic variables. The research ethics committee of Kozminski University reviewed the survey and approved it on 4 May 2023. The database and the survey questionnaire are publicly accessible via the link https://figshare.com/articles/dataset/Transparency_Sunshine_Act_Poland/24598488 accessed on 19 December 2024.

Each participant’s consent to participate in the study was obtained electronically. Only those who agreed to participate after being informed of the study’s purpose and methods had access to the survey.

### 2.4. Queries and Variables

This paper analyzes the responses to 15 statements (listed in Table 2), rated by the respondents on a scale of 1–10, with 1 indicating strong disagreement and 10 indicating strong agreement. Cronbach’s alpha coefficient for the analyzed questions was found to be 0.744, indicating an acceptable level of reliability. The normality of the frequency distributions of the responses to individual statements tested using Shapiro–Wilk tests and histogram analysis demonstrated a lack of a normal distribution. Consequently, Mann–Whitney U-tests were conducted to evaluate the statistical relationships between statements about the expectations of the GPs and socio-demographic characteristics, particularly in instances where there were two distinct values for demographic variables. In cases where the variables exhibited more than two values, the Kruskal–Wallis H-test was employed. In addition, Spearman’s rho correlation was used for continuous variables. The trust in GPs was measured by the question, “In general, do you trust GPs?” where 1 means not at all and 10 means very much.

## 3. Results

**Figure 1:**
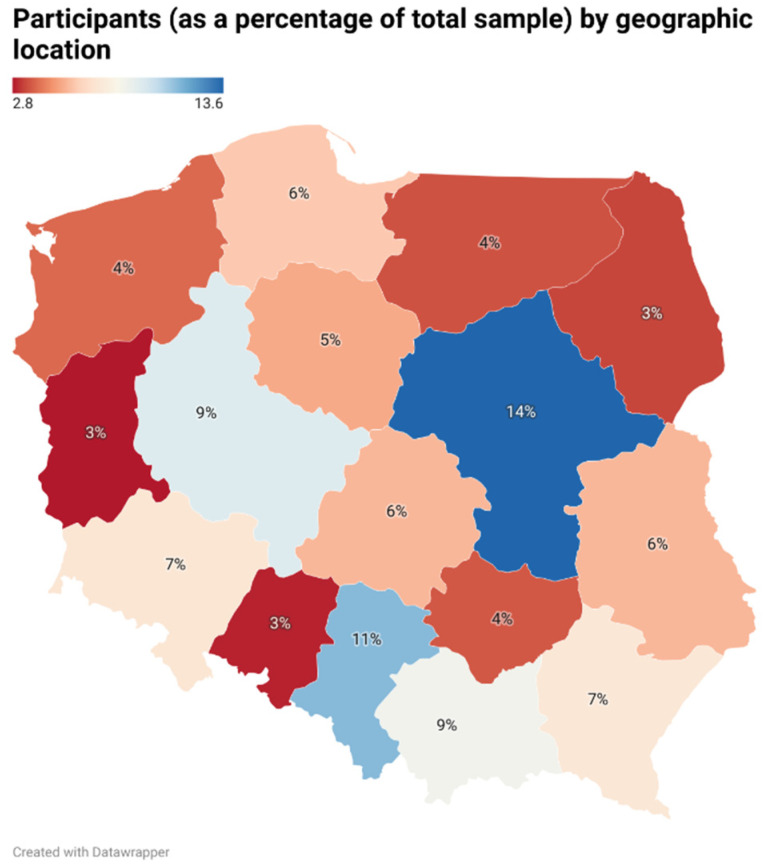
The survey participants were distributed nationwide, reflecting the population density per voivodship (Polish province).

A total of 1057 respondents representing all 16 voivodships (Polish provinces; Figure 1) satisfactorily completed the questionnaire, providing accurate and comprehensive responses. The initial survey cohort comprised 1497 individuals, of whom 440 were excluded on the basis of the criteria stated in Section 2.1.

The survey participants wanted their GPs to remain educated throughout their careers and provide honest feedback, even if unfavorable. The two items (#1 and #2; see Table 2) representing these attributes received the highest mean value (M = 8.6) among all the statements included in the survey. Subsequently, the survey participants preferred GPs to facilitate patients’ comprehension of their condition. Furthermore, the participants wanted GPs to avoid displaying a sense of superiority. Both statements had a mean value of 8.5. The additional preferences of the survey participants are delineated in Table 2.

Attributes that are essential in characterizing the optimal GP’s care varied by age group. The youngest respondents (18–24 years) assigned the least importance among the age groups to the GP’s education (Item #1), truthfulness (Item #2), ability to convey information (Item #3), kindness (Items #4 and 9), encouragement of lifestyle changes (Item #5), and concern for patients’ well-being (Items #6 and #8). Furthermore, the GP’s listening skills (Item #7), empathy (Items #10 and #13), knowledge of their patients (Item #12), or the GP’s acceptance of gifts (Item #14) were less critical to the youngest respondents than to the other age groups. The youngest participants also had greater acceptance of discussing personal or professional problems with their GP than other age groups. However, the opinion of the youngest respondents regarding the GP’s respect for the patients’ reluctance to discuss an embarrassing topic (Item #11) was similar to that of the other age groups. Particularly significant differences can be seen when the group of the youngest respondents is compared with the oldest (Table 2).

Using the continuous age variable resulted in a statistically significant correlation between age and most statements. Spearman’s rho correlation analysis indicated relationships between age and most statements, except for Items #11 and #15. However, the value of the rho coefficient shows that these correlations are relatively weak. The strongest correlation between age and Item #12 was observed, indicating that the patients’ wish for GPs to recognize their regular patients and know their medical conditions significantly increased with age (Table 2).

The mean level of trust in GPs was 6.8 (median = 7). There was a significant correlation between the value of individual claims and trust in GPs (Table 2), although, according to Akoglu [12], they should be considered weak.

Moreover, we examined the correlation between healthcare utilization over the past six months and perceptions of the attributes of an exemplary GP (Table 3). The findings indicated that for 14 of the 15 statements, healthcare users demonstrated statistically significantly higher expectations. The sole exception was the statement, “The GP should not accept patient gifts as a sign of gratitude”, for which the result remained consistent regardless of the frequency of healthcare utilization.

**Figure 2:**
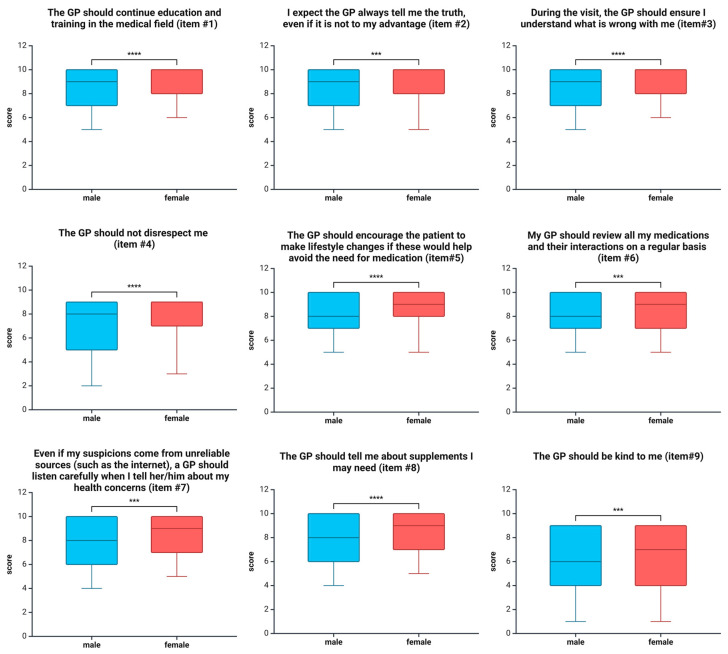
Overview of items yielding significant differences between women and men. The boxplot (5–95 percentile) presents the scores in response to Items #1–9. The non-binary survey participants were excluded from this comparison due to the small sample size (*n* = 2). The significance of differences between groups was calculated using the Kruskal–Wallis test with Dunn’s multiple comparisons test. *** *p* < 0.0001; **** *p* < 0.00001.

Furthermore, our findings suggest that women have higher expectations of GPs than men. As illustrated in Figure 2, Items #1–9 yielded higher values for women than men. No significant differences were seen for Items #10–15. However, two instances were observed where their expectations were slightly lower. Firstly, male respondents indicated a stronger preference for comfort. Secondly, men agreed more that their private and professional lives should be discussed with a medical practitioner. Nevertheless, the observed differences between the sexes were insignificant in both cases.

Individuals who are satisfied with their quality of life are more likely to seek guidance and information from a well-educated (Item #1) GP than those who are dissatisfied. Moreover, they are more likely than those who are dissatisfied to advocate the view that GP should always be truthful with patients (Item #2), ensure that patients comprehend the situation they are in (Item #3), and inform them about the recommended dietary supplements (Item #8). Furthermore, they are more likely to believe that a GP should encourage the patient to make lifestyle changes if they can avoid taking medications (Item #5) and check for interactions between the patient’s medications (Item #6).

Significant discrepancies were observed between groups with different self-rated health. The group comprising individuals who rated their health as “bad and very bad” exhibited a statistically significant tendency to desire that GP convey even the most unfavorable assessment (Item #2), review the patient’s medications (Item #6), provide information about dietary supplements (Item #8), ascertain the patient’s regular medical providers and their medical history (Item #12), and demonstrate empathy through physical contact, such as touching the patient’s hand or arm (Item #10).

Furthermore, the discrepancies between the respondents’ education level and perceptions of the GP’s role were investigated. Notably, this socio-demographic characteristic was found to differentiate the respondents for only five statements. Specifically, the statements included the following recommendations, namely that the GP should engage in lifelong learning (Item #1), should treat the patient with respect (Item #4), and should encourage the patient to modify their lifestyle if doing so could help avoid the use of drugs (Item #5), should monitor for potential drug interactions (Item #6), and should refrain from accepting gifts from patients (Item #14). The group with the highest level of education demonstrated the most substantial expectations regarding each of these statements.

Moreover, we aimed to ascertain whether there was a statistically significant relationship between the utilization of healthcare services over the past six months and the socio-demographic attributes of the respondents. Such a relationship was identified concerning sex (female respondents utilized healthcare services more frequently), self-rated health status (those with the poorest self-rated health status utilized healthcare services most frequently), and education (respondents with the highest level of education utilized healthcare services most frequently). A detailed account of these findings can be found in Table 4. Additionally, healthcare users exhibited greater trust in GPs (Mann–Whitney U = 109.5, df = 1, *p* = 0.003). The survey showed a relationship between the age of the participants and the frequency of healthcare services used six months before the survey. Among the youngest people (18–24), 54.4% used medical services; in the 25–34 group, 57.9%; in the 35–44 group, 65.9%; in the 45–54 group, 69%; and in the 55–54 group, 79.8%.

## 4. Discussion

The objective of our study was to ascertain which attributes are most highly valued by the general adult population concerning GPs. Our findings revealed that age constituted a significant socio-demographic factor influencing the expectations of GPs. The 55+ age group exhibited the highest expectations, while the youngest (18–24) demonstrated the lowest expectations. In only two cases—statements about the GP asking embarrassing questions and discussing private and professional issues—no correlation indicated that those patients’ expectations change with age. This could be attributed to factors such as educational attainment, cultural shifts, and the increased visibility of health-related discourse in the media.

Additionally, our findings indicate an increase in the prevalence of medical care utilization with age. A greater proportion of individuals in older age groups had utilized healthcare services in the past six months than individuals in younger age groups. A recent CBOS survey yielded comparable findings, indicating that the youngest cohort in Poland exhibited the lowest prevalence of healthcare utilization over the past six months [13]. The conjunction of these two findings leads to the conclusion that as individuals age, there is an increase in the frequency of their contact with medical professionals. Likewise, studies from other countries indicate that older individuals are the most frequent users of healthcare services [13] and are more likely to have at least one chronic condition [14] that necessitates continuous medical attention. Therefore, our study demonstrates that adequate medical care becomes increasingly critical as individuals age. While the increased utilization of healthcare services influences this, it is also shaped by the indirect experience of providing care for others (e.g., children or parents). Young individuals do not encounter this challenge to the same extent. Additionally, women, who are more likely to assume a caregiving role within the family, are more likely to bear such responsibilities than men [15,16].

Furthermore, our findings indicate that women have higher expectations of GPs than men. Women allocated significantly higher values for most statements than men (Items #1–9; see Table 2). The existing literature provides ample evidence that women often demonstrate higher levels of empathy than men [17]. They tend to seek more empathetic listening and extended visits, mainly when consulting with female physicians [18]. Additionally, during visits, women are more likely to raise emotional issues [19].

Furthermore, the data indicated that women were more likely than men to have utilized healthcare services within the past six months. The phenomenon known as the “male–female health–survival paradox” posits that women tend to experience higher rates of illness, disability, and lower self-rated health compared with men. However, women consistently demonstrate lower mortality rates and enjoy greater life expectancy across most developed countries [16,20]. Female patients are more likely to have multiple medical appointments and incur higher annual medical expenses [21,22]. A recent Australian study demonstrated that the utilization patterns of health services among men are predominantly influenced by age-related factors [23]. A consistent pattern emerges, indicating that men do not engage sufficiently in routine preventive healthcare services and delay seeking assistance until treatment options are limited [24,25].

The mean level of trust in GPs was 6.8, with a median value of 7. According to the IPSOS Global Trustworthiness Report, Poland is among the countries with the lowest levels of trust in medical professionals [14]. A substantial body of prior research provides evidence to support this assertion [26]. Research shows that trust significantly influences health-promoting behaviors [27,28]. Our present study revealed a statistically significant relationship between trust in medical doctors and the utilization of healthcare services within the past six months. Consequently, medical professionals should prioritize fostering high levels of trust among their patients, which can be achieved, for example, through effective communication [29]. Our survey results indicate a correlation between trust in GPs and the majority of selected statements. This suggests that as trust in GPs increases, so do the expectations. However, it is essential to note that most identified correlations were relatively weak. It may be hypothesized that if GPs meet the patients’ expectations, patients will subsequently demonstrate greater trust in them. However, this relationship was not examined in the present study, and further investigation is required. Interestingly, trust in GPs did not correlate with patients’ readiness to discuss personal and professional problems. These findings also require verification and deeper analysis in future studies.

The data indicated that there were only a few statistically significant differences in the respondents’ ratings of desirable GP characteristics according to their level of life satisfaction. Those who reported high levels of life satisfaction rated higher on statements regarding the importance of educating the GP and informing the patient about various issues. These two domains are interrelated: a more highly educated GP is more competent and better positioned to inform the patient. Medicine constantly evolves, necessitating physicians to maintain an up-to-date knowledge base [30]. Medical practitioners are more inclined to pursue further education if they know that at least one of their patients will benefit from their continuing education [31]. However, our findings revealed no statistically significant relation between life satisfaction and healthcare utilization within the past six months. Our research suggests that individuals who report high levels of life satisfaction tend to rate their health status more favorably than those who are less satisfied. Corroborating this observation, studies conducted in diverse populations and contexts consistently demonstrate a positive association between life satisfaction and self-assessed health status [32,33].

Assessing one’s health may diverge from the objective reality of one’s health status. However, it has been demonstrated that self-assessment correlates with clinically measured health assessment and can serve as a valuable indicator [34]. Individuals who rate their health more poorly are also likely to require more significant support from their GPs. This support may take the form of listening, empathy, and understanding. The results of our study can be explained by demonstrating that individuals who rated their health most poorly on many of the statements we examined received the highest mean ranks, suggesting that they expect the most from their doctors. However, statistically significant differences among those who rated their life satisfaction differently occurred for several statements, primarily concerning the fact that individuals with poor health had higher information expectations and were statistically significantly more likely to expect their doctors to remember their regular patients and to allow the doctor to touch their hand or arm. It has long been established that individuals experiencing illness require physical contact, a form of care that their healthy family members and friends often avoid due to concerns about infection [35]. It is logical to conclude that individuals with poorer self-rated health were the most likely to have visited a healthcare professional in the previous six months. This is consistent with the observation that those with the poorest health are also the most frequent users of healthcare services [36].

People with more than a high school education used healthcare services more often than those without, supporting the view that education leads to more frequent use of preventive care and health services [37,38]. Better-educated individuals often held more advanced expectations of medical professionals than those with less education. They likely expect their GP to have extensive medical knowledge. Furthermore, they are aware of the risks associated with inappropriate medications. Moreover, they expect greater respect from the GP. One might hypothesize that they perceive the knowledge asymmetry between themselves and the GP to be less pronounced. Finally, they are less tolerant of GPs accepting gifts from patients (up to 11% of Polish people have offered gifts to medical professionals in the past four to five years [39]), confirming that education affects tolerance of corruption [40].

The principal limitation of the study is the use of an online survey technique, which remains reliant on quota sampling. Although the study reflects the social structure of Polish society, it cannot be considered representative, as a significant proportion of people, especially older adults, still do not use the internet in Poland [13]. As a result, it is not possible to generalize the findings to the entire Polish population; instead, they can only be generalized to internet users. A further limitation is that individuals with poor health were unlikely to complete the online survey. In our sample, only a small proportion of respondents described their health as poor or very poor, which may have influenced the results. Further research on this group would be beneficial, for instance, through in-depth interviews, to gain a deeper understanding of their opinions. It is important to note that self-assessment of health status is only one indicator of the patient’s overall health status. It is essential to consider objective health indicators, such as the patient’s medical condition, to better understand their health status. Nevertheless, obtaining such an indicator in patient surveys is not feasible. To gain a more accurate understanding of the patient’s health status, it would be necessary to have access to their medical records.

## 5. Conclusions

In Poland, higher expectations of a physician’s attention to humanizing aspects seem to increase with the age of respondents and the more trust they have in doctors. Women also have higher expectations than men, as do those who are satisfied with their lives and those who are less satisfied with their health. It is essential for physicians to be aware of these differences and to tailor their approach and services to the individual needs and expectations of their patients. In doing so, they can provide more effective and satisfying medical care that better meets the diverse needs of the patient community. It is similarly vital to disseminate the acquired knowledge to the medical undergraduates enrolled in medical humanization courses. This will equip them with the requisite skills to meet future demands.

## Figures and Tables

**Table 1:** Sample characteristics.

Socio-Demographic Characteristics of the Sample		n	%
Gender	Female	558	52.8
	Male	497	47.0
	Non-binary	2	0.2
Age	18–24 years old	103	9.7
	25–34 years old	221	20.9
	35–44 years old	170	16.1
	45–54 years old	200	18.9
	55–64	186	17.6
	65 years old and more	177	16.7
Educational level	Primary	51	4.8
	Gymnasium	17	1.6
	Vocational	153	14.5
	Secondary	574	54.3
	Bachelor’s degree	83	7.9
	Master’s degree	167	15.8
	Doctorate and upper	12	1.1
Size of the place of residence	Village	399	37.7
	City up to 20,999 residents	139	13.2
	City 21,000–100,999 residents	200	18.9
	City 101,000–500,999 residents	183	17.3
	City with over 501,000 residents	136	12.9

**Table 2:** Desirable qualities of a GP as perceived by patients in different age groups (N = 1057).

Survey Items (Statements):	Total Mean (SD)	Total Median (Q1–Q3)	Mean Rank in the Age Groups (*n*); Age Reported in Year Ranges						Age (Continuous M = 46.5; Mdn = 46)	Trust in GP (M = 6.8; Mdn = 7)
			18–24 (103)	25–34 (221)	35–44 (170)	45–54 (200)	55+ (363)	Kruskal–Wallis H (df) *p*	Spearman’s Rho *p*	Spearman’s Rho *p*
1. The GP should continue education and training in the medical field.	8.6 (1.7)	9 (8–10)	397.2	474.6	514.0	540.3	600.3	52.6 (4) *p* < 0.001	0.2 *p* < 0.001	0.2 *p* < 0.001
2. I expect the GP always tell me the truth, even if it is not to my advantage.	8.6 (1.8)	9 (8–10)	440.1	506.8	524.9	533.9	567.0	17.5 (4) *p* = 0.002	0.1 *p* < 0.001	0.2 *p* < 0.001
3. During the visit, the GP should ensure I understand what is wrong with me.	8.5 (1.8)	9 (8–10)	417.1	476.8	506.6	555.5	588.5	39.7 (4) *p* < 0.001	0.2 *p* < 0.001	0.2 *p* < 0.001
4. The GP should not disrespect me. *	8.5 (2.2)	10 (7–10)	448.2	459.3	516.3	570.3	577.5	37.4 (4) *p* < 0.001	0.1 *p* < 0.001	0.0
5. The GP should encourage the patient to make lifestyle changes if these would help avoid the need for medication.	8.2 (1.8)	9 (7–10)	375.8	481.7	494.6	566.1	596.9	57.8 (4) *p* < 0.001	0.2 *p* < 0.001	0.2 *p* < 0.001
6. My GP should review all my medications and their interactions on a regular basis.	8.1 (1.9)	8 (7–10)	365.6	476.7	508.6	522.5	620.4	73.3 (4) *p* < 0.001	0.3 *p* < 0.001	0.1 *p* < 0.001
7. Even if my suspicions come from unreliable sources (such as the internet), a GP should listen carefully when I tell her/him about my health concerns.	8.1 (1.9)	8 (7–10)	404.2	484.9	509.3	533.5	598.0	43.1 (4) *p* < 0.001	0.2 *p* < 0.001	0.1 *p* < 0.001
8. The GP should tell me about supplements I may need (e.g., prebiotics, magnesium, vitamin D).	8.0 (2.0)	8 (7–10)	416.6	518.3	514.4	544.6	565.7	21.4 (4) *p* < 0.001	0.1 *p* < 0.001	0.2 *p* < 0.001
9. The GP should be kind to me. *	7.1 (2.8)	8 (5–10)	484.7	495.0	521.5	544.0	557.5	9.0 (4) *p* < 0.061	0.1 *p* < 0.006	0.0
10. If the GP gives me negative information, she/he may touch my arm or hand.	6.5 (2.6)	7 (5–8)	366.3	459.6	498.4	542.5	624.3	79.5 (4) *p* < 0.001	0.3 *p* < 0.001	0.2 *p* < 0.001
11. The GP should not continue to ask if the patient is embarrassed to tell. *	6.5 (2.7)	7 (5–9)	526.5	537.8	525.6	529.2	525.9	0.25 (4) *p* = 0.993	0.0	0.0
12. A GP should know all their regular patients and their medical conditions.	6.4 (2.4)	6 (5–8)	338.7	413.6	504.1	568.4	643.2	129.0 (4) *p* < 0.001	0.3 *p* < 0.001	0.1 *p* < 0.001
13. The GP should comfort me when my health is not good.	6.3 (2.3)	6 (5–8)	427.5	461.6	508.0	557.6	592.9	41.4 (4) *p* < 0.001	0.2 *p* < 0.001	0.2 *p* < 0.001
14. The GP should not accept patient gifts as a sign of gratitude. *	6.2 (2.8)	6 (4–9)	471.4	485.0	558.8	567.6	536.9	13.6 (4) *p* = 0.009	0.1 *p* = 0.013	0.2 *p* < 0.001
15. My personal or professional problems should be discussed with the GP. *	4.6 (2.7)	5 (2–6)	579.1	513.9	518.0	503.1	543.4	5.9 (4) *p* = 0.208	0.0	0.0

* These statements have been reversed to represent the desired quality of the GP instead of stating undesirable qualities.

**Table 3:** Desired characteristics of a GP among patients stratified by recent healthcare usage, gender, life satisfaction, self-rated health, and education (N = 1057).

	Healthcare USAGE in the Last Six Months			Life Satisfaction (*n* ^1^), Mean Rank			Self-Assessment of Health (n), Mean Rank				Education (n), Mean Rank			
	Yes (709)	No (348)	Mann–Whitney U *p*	Satisfied (756)	Unsatisfied (163)	Mann–Whitney U *p*	Very Good and Good (474)	Moderate (471)	Bad and Very Bad (96)	Kruskal–Wallis H (df) *p*	Lower than Secondary (221)	Secondary (574)	Higher than Secondary (262)	Kruskal–Wallis H (df) *p*
1. The GP should continue education and training in the medical field.	551.8	482.5	107,171 *p* < 0.001	470.3	412.5	53,868 *p* = 0.07	511.5	519.8	573.8	3.9 (2) *p* = 0.114	495.9	523.6	568.7	8.1 (2) *p* = 0.02
2. I expect the GP always tell me the truth, even if it is not to my advantage.	554.9	476.2	104,995 *p* < 0.001	469.8	414.6	54,270 *p* = 0.01	530.3	498.2	586.9	8.7 (2) *p* = 0.013	529.1	527.1	533.0	0.1 (2) *p* = 0.936
3. During the visit, the GP should ensure I understand what is wrong with me.	551.2	483.7	107,604 *p* < 0.001	476.2	385.1	49,398 *p* < 0.001	522.3	514.9	544.6	0.9 (2) *p* = 0.648	494.2	533.3	549.0	4.5 (2) *p* = 0.107
4. The GP should not disrespect me. *	550.3	485.6	108,272 *p* < 0.001	465.5	434.4	57,438 *p* = 0.14	517.4	523.0	528.8	0.2 (2) *p* = *0*.914	484.2	525.5	574.3	12.5 (2) *p* = *0*.002
5. The GP should encourage the patient to make lifestyle changes if these would help avoid the need for medication.	556.3	473.4	104,010 *p* < 0.001	474.2	394.2	50,880 *p* < 0.001	533.7	499.9	561.8	5.3 (2) *p* = *0*.072	486.1	523.1	578.0	12.0 (2) *p* = *0*.002
6. My GP should review all my medications and their interactions on a regular basis.	554.2	477.7	105,510 *p* < 0.001	474.1	394.7	50,977 *p* < 0.001	525.7	502.5	588.4	7.1 (2) *p* = 0.029	484.9	534.4	554.4	7.0 (2) *p* = 0.03
7. Even if my suspicions come from unreliable sources (such as the internet), a GP should listen carefully when I tell her/him about my health concerns.	558	470	102,828 *p* < 0.001	466.9	427.9	56,385 *p* = 0.081	510.1	527.5	543.0	1.4 (2) *p* = 0.492	498.5	532.3	547.4	3.4 (2) *p* = 0.184
8. The GP should tell me about supplements I may need (e.g., prebiotics, magnesium, vitamin D).	555.2	475.7	104,803 *p* < 0.001	472.3	402.9	52,309 *p* = 0.002	534.5	497.1	571.3	6.9 (2) *p* = 0.031	504.6	532.4	542.2	2.1 (2) *p* = 0.355
9. The GP should be kind to me. *	551.5	483.2	107,414 *p* < 0.001	460.0	460.1	61,596 *p* = 0.995	512.6	524.9	543.1	1.0 (2) *p* = 0.606	491.8	538.0	540.6	4.3 (2) *p* = 0.115
10. If the GP gives me negative information, she/he may touch my arm or hand.	550.3	485.7	108,295 *p* = 0.001	467.5	425.2	55,938 *p* = 0.063	508.4	517.6	599.6	7.6 (2) *p* = 0.023	567.9	517.7	520.9	4.6 (2) *p* = 0.098
11. The GP should not continue to ask if the patient is embarrassed to tell. *	544.7	496.9	112,210 *p* = 0.016	463.7	442.7	58,787 *p* = 0.354	544.8	499.1	510.6	5.7 (2) *p* = 0.059	516.6	516.2	567.6	5.7 (2) *p* = 0.059
12. A GP should know all their regular patients and their medical conditions.	550.9	484.4	107,844 *p* < 0.001	462.4	449.0	59,814 *p* = 0.555	504.4	517.1	621.9	12.6 (2) *p* = 0.002	520.3	541.7	508.4	2.4 (2) *p* = 0.3
13. The GP should comfort me when my health is not good.	546.8	492.8	110,764 *p* = 0.006	459.4	462.9	61,140 *p* = 0.876	509.4	527.0	548.8	1.7 (2) *p* = 0.419	516.5	547.4	499.2	5.0 (2) *p* = 0.08
14. The GP should not accept patient gifts as a sign of gratitude. *	519.2	548.9	116,433 *p* = 0.134	456.0	478.6	58,577 *p* = 0.319	511.0	533.0	511.3	1.4 (2) *p* = 0.498	484.7	528.7	567.1	8.9 (2) *p* = 0.012
15. My personal or professional problems should be discussed with the GP. *	541.9	502.7	114,222 *p* = 0.048	453.3	491.2	56,523 *p* = 0.095	512.1	535.6	493.3	2.4 (2) *p* = 0.304	526.4	532.5	523.6	0.2 (2) *p* = 0.916

* These statements have been reversed to represent the desired quality of the GP instead of stating undesirable qualities. ^1^ Answers of “hard to say” were eliminated from this particular analysis.

**Table 4:** The utilization of health services by the sample respondents in the six months preceding the survey, with a particular focus on the relationship between this utilization and the respondents’ socio-demographic characteristics.

Healthcare Usage in the Last Six Months	Sex (*n*)			Life Satisfaction (*n*)			Self-Assessment of Health (*n*)				Education (*n*)			
	Male (497)	Female (558)	Ch^2^(df) *p*	Satisfied (756)	Unsatisfied (163)	Ch^2^(df) *p*	Very Good and Good (474)	Moderate (471)	Bad and Very Bad (96)	Ch^2^(df) *p*	Lower than Secondary (221)	Secondary (574)	Higher than Secondary (262)	Ch^2^(df) *p*
Yes (709)	73.7%	59.6%	23.6 (1) *p* < 0.001	68.3%	66.9%	0.1 (1) *p* = 0.731	59.1%	72.8%	79.2%	21.2 (2) *p* < 0.001	58.4%	67.1%	74.4%	14.0 (2) *p* < 0.001
No (348)	26.3%	40.4%		31.7%	33.1%		40.9%	27.2%	20.8%		41.6%	32.9%	25.6%	

## Data Availability

The database and the survey questionnaire are publicly available via the link https://figshare.com/articles/dataset/Transparency_Sunshine_Act_Poland/24598488 (accessed on 24 October 2024).
